# Laparoscopic Heller myotomy and Dor fundoplication following an unsuccessful peroral endoscopic myotomy

**DOI:** 10.1186/s40792-023-01691-y

**Published:** 2023-06-14

**Authors:** Takuma Aoki, Soji Ozawa, Koki Hayashi, Tomofumi Ando, Yusuke Uchi, Motohide Shimazu, Hiroharu Shinozaki, Kenji Matsumoto, Nobuo Omura

**Affiliations:** 1 Department of Surgery, Tamakyuryo Hospital, 1491 Shimooyamada, Machida, Tokyo 194-0297 Japan; 2 Department of Surgery, Saiseikai Utsunomiya Hospital, 911-1 Takebayashi, Utsunomiya, Tochigi 321-0974 Japan; 3 Department of Surgery, National Hospital Organization Nishisaitama‐Chuo National Hospital, 2‐1671 Wakasa, Tokorozawa, Saitama 359-1151 Japan

**Keywords:** Achalasia, Heller–Dor, Per-oral endoscopic myotomy

## Abstract

**Background:**

Achalasia is an esophageal motility disorder that presents as dysphagia and severely affects quality of life. An esophageal myotomy has been the golden standard for treatment. Peroral endoscopic myotomy (POEM) as a first-line therapy has an acceptable outcome. However, after the clinical failure of POEM, appropriate second-line therapy is rather controversial. Here, we present the first published case in English of a patient who was successfully treated using laparoscopic Heller myotomy (LHM) with Dor fundoplication following an unsuccessful POEM.

**Case presentation:**

A 64-year-old man with type 1 achalasia who had been previously treated with POEM visited our hospital for further treatment. After undergoing LHM with Dor fundoplication, his Eckardt score improved from 3 to 0 points. On a timed barium esophagogram (TBE), the barium height improved from 119 mm/119 mm (1 min/5 min) to 50 mm/45 mm. No significant complications have occurred postoperatively for 1 year.

**Conclusion:**

Treating refractory achalasia is challenging, and treatment options are controversial. LHM with Dor fundoplication after POEM could be a safe and efficient option for the treatment of refractory achalasia.

## Introduction

Esophageal achalasia was first described in 1674 by Sir Thomas Willis as a constellation of dysphagia, the regurgitation of undigested food, respiratory symptoms, chest pain, and weight loss [1]. The disease prevalence is assumed to be 1 in 100,000 individuals [2]. From a pathophysiological perspective, esophageal achalasia has been described as a degeneration of the myenteric neurons of the lower esophageal sphincter (LES) [2]. Achalasia impairs patients’ quality of life and is incurable. Attaining a tolerable level of remission is the key to treatment. Treatment options include pneumatic dilation, botulinum toxin injection, myotomy, and medical therapy. A myotomy can be performed endoscopically or surgically. While all these options are essentially palliative, surgical myotomy, such as a laparoscopic Heller myotomy (LHM) with fundoplication, has been the golden standard for treatment with the best clinical success rate. The introduction of per-oral endoscopic myotomy (POEM) in 2007 enabled acceptable outcomes with a lower degree of invasiveness, and POEM has become a preferred treatment option [3, 4]. Presently, POEM is performed as the treatment of first choice at institutions where POEM is a feasible option [5]. However, even though the results of POEM are generally acceptable, the treatment of some patients can be incomplete [6]. Additional intervention is required for patients with incomplete POEM, and second-line options remain a matter of debate. Repeat POEM and LHM are both mainstream treatments following unsuccessful POEM. The efficacy and safety of these second-line procedures have been discussed in several papers previously. However, data and evidence remain insufficient because of the rarity of this condition. Thus, the sharing of treatment experiences and the provision of detailed clinical data are essential. This report is the first English publication describing an LHM with Dor fundoplication procedure following an unsuccessful POEM.

## Case presentation

A 64-year-old man presented with dysphagia at a high-volume center for POEM in Japan. His previous medical history was of minimal significance except for the presence of well-controlled type 2 diabetes mellitus. He underwent POEM, which decreased his Eckardt score from 8 to 3 points. His symptoms were not alleviated to the expected extent, and he continued to suffer when eating solid foods, such as beef steak. He spontaneously visited our hospital for further surgical treatment 2 years after undergoing his initial POEM procedure.

**Fig. 1:**
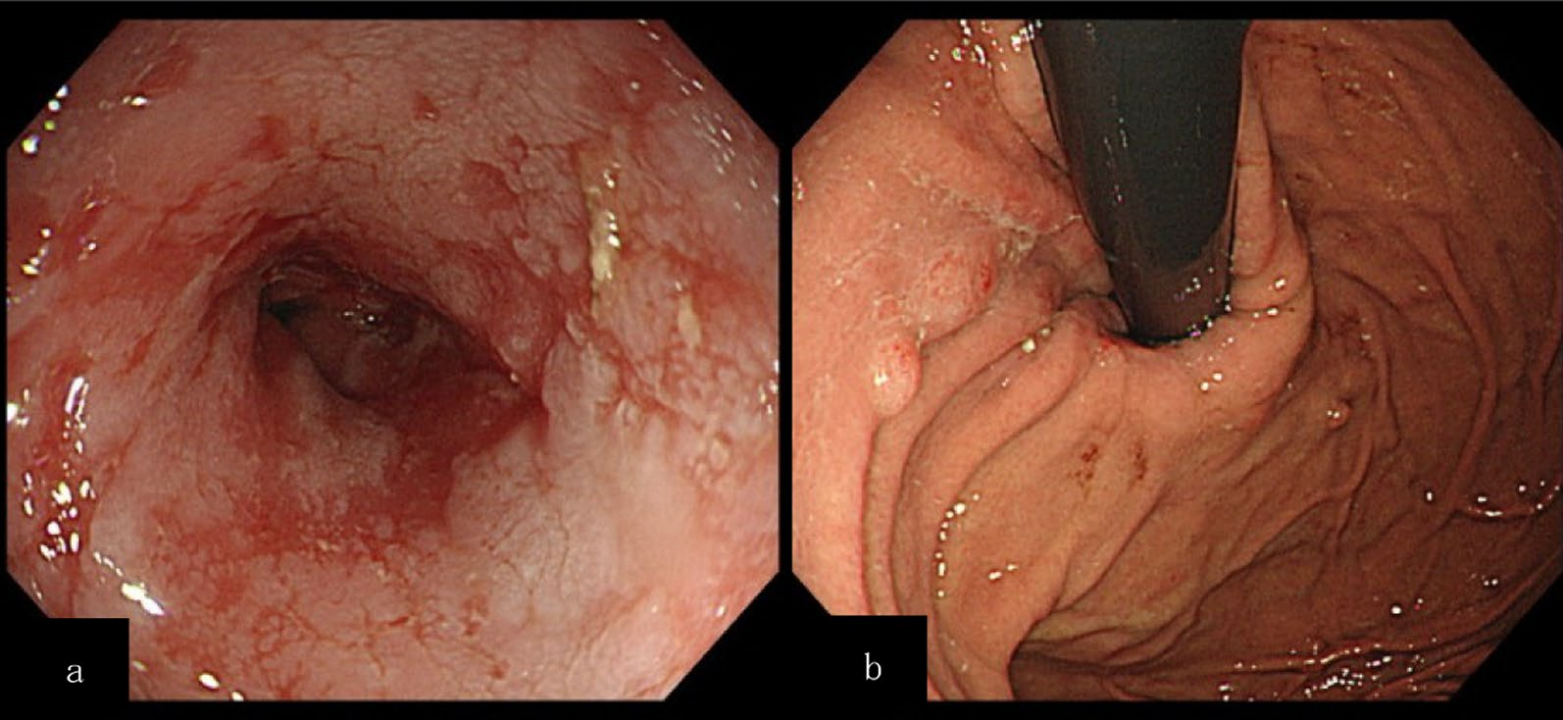
Esophagogastroduodenoscopy findings before the second treatment. **a** Los Angeles classification: Grade B. **b** Cardia before fundoplication

**Fig. 2:**
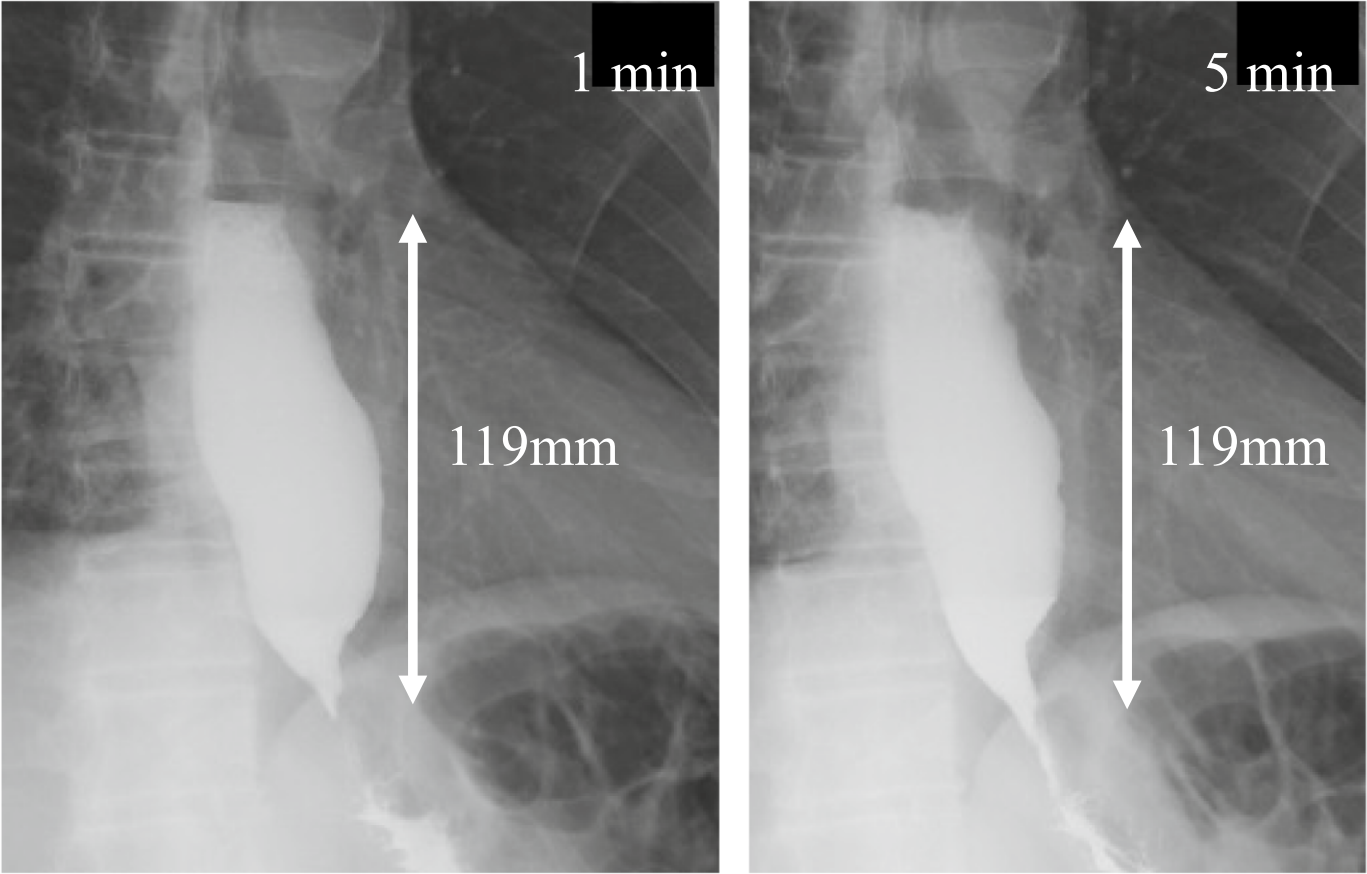
Timed barium esophagogram results before the second treatment: 119 mm in height at 1 min, and 119 mm at 5 min

Preoperative examinations were performed. An esophagogastroduodenoscopy (EGD) showed Grade B reflux esophagitis with long-segment Barrett’s esophagus (Fig. 1). A biopsy ruled out malignancy. A timed barium esophagogram (TBE) resulted in barium column heights at 1 min and 5 min of 119 mm and 119 mm, respectively (Fig. 2). Computed tomography showed an apparent dilation of the thoracic esophagus with no signs of malignancy. A 24-h esophageal multichannel intraluminal impedance and pH monitoring examination showed an acid exposure time (AET) of 8.2% and a DeMeester score of 24.5, indicating morbid reflux. High-resolution manometry (HRM) revealed a normal integrated relaxation pressure (IRP) of 13.6 mmHg and the absence of a peristaltic wave. These results were compatible with a diagnosis of Chicago type 1 achalasia complicated by post-POEM reflux esophagitis. Considering the lack of LES relaxation and the delayed esophageal outflow, the prior POEM was deemed to have been incomplete. The laboratory data showed no abnormalities, including tumor markers. As the patient was a candidate for an additional POEM, a repeat POEM was recommended [7]. However, the patient refused to undergo a repeat POEM because of a profound apprehension of another failure.

**Fig. 3:**
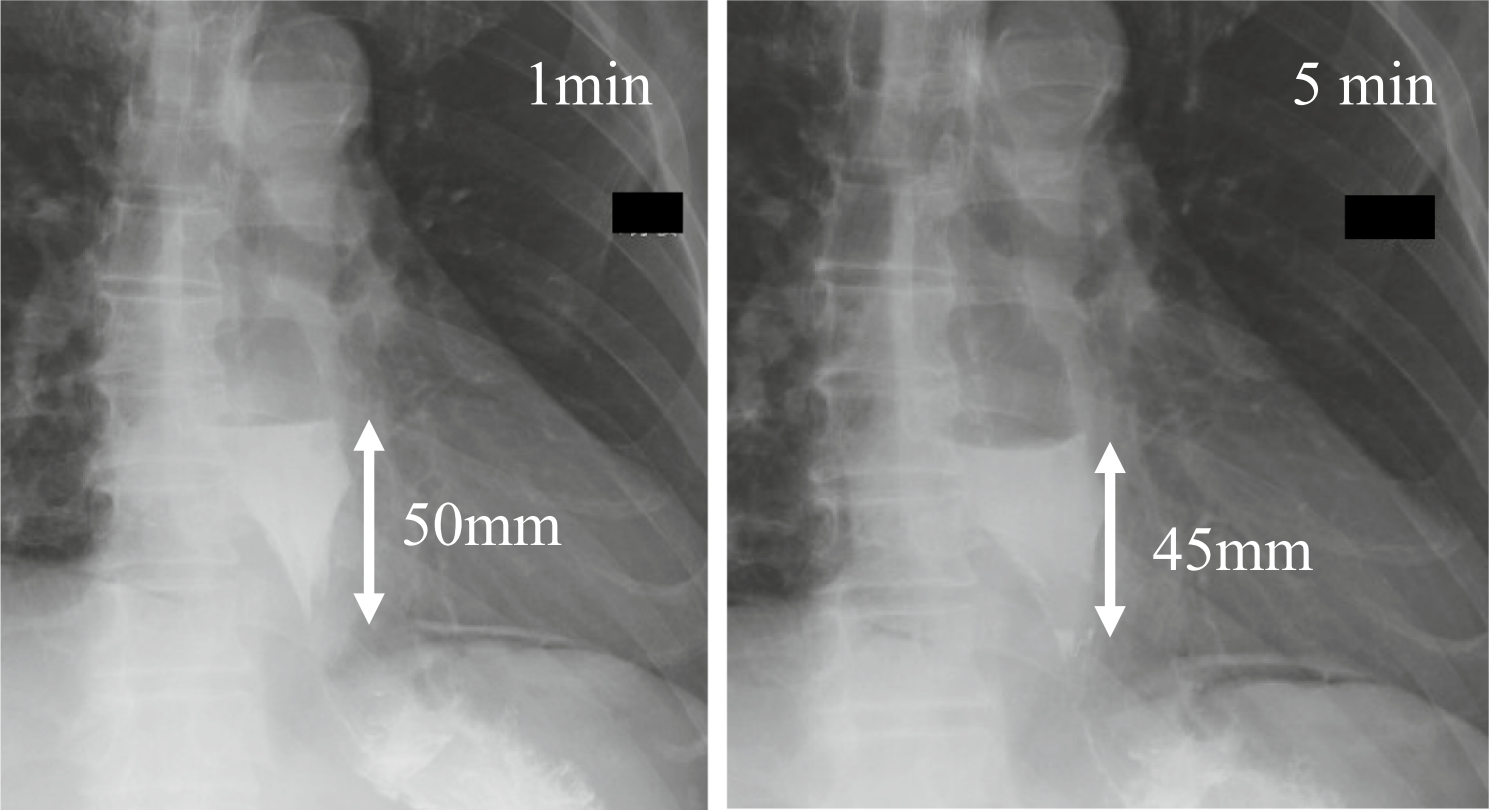
Timed barium esophagogram results after the second treatment: 50 mm in height at 1 min, and 45 mm at 5 min

**Fig. 4:**
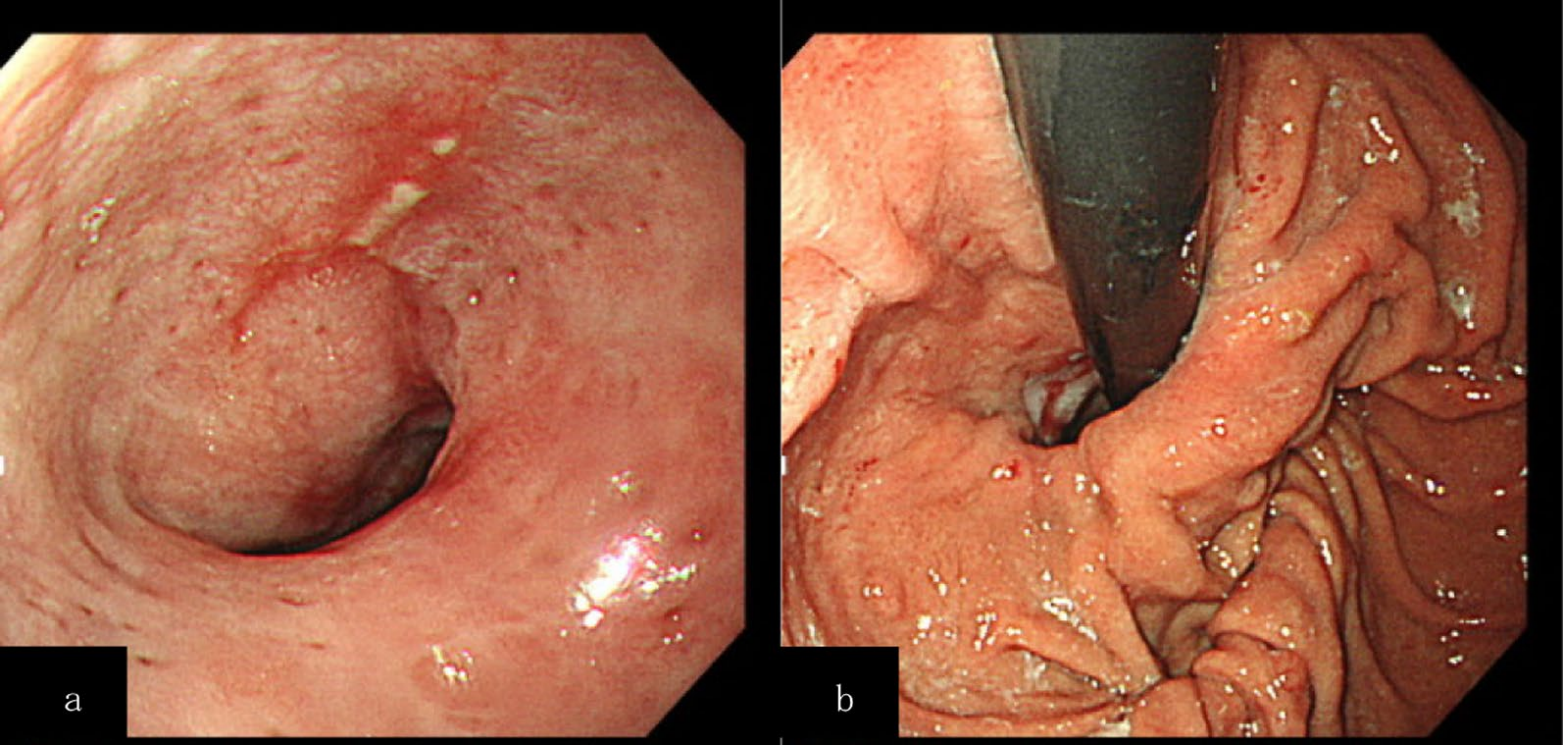
Esophagogastroduodenoscopy findings after the second treatment. **a** Los Angeles classification: Grade A. **b** Cardia after fundoplication

After a thorough preoperative examination and evaluation, he underwent LHM with Dor fundoplication. Neither the previous doctor nor we prescribed proton pump inhibitors (PPIs) prior to the surgery. The operation was performed by an experienced surgeon using 5 ports. Some fibrotic changes in the posterior esophageal wall were present, possibly because of the prior POEM. As the chief complaint was difficulty swallowing, the fundoplication was performed in a manner that would guarantee food passage. The myotomy was completed 4 cm above and 2 cm below the esophagogastric junction. The operating time was 242 min, and the operative blood loss was 5 mL. The postoperative course was uncomplicated. Oral intake was resumed on postoperative day 2 after a TBE examination. The TBE barium height had improved to 50 mm/45 mm (Fig. 3). He was discharged on postoperative day 5 with vonoprazan 10 mg for prophylaxis of GERD. EGD showed improvement in reflux esophagitis (Fig. 4). At a 7-month postoperative visit to our outpatient clinic, he was asymptomatic and had an Eckardt score of 0 points.

## Discussion

We treated a patient with type 1 achalasia who was successfully managed using LHM with Dor fundoplication as a salvage therapy after an unsuccessful POEM. The postoperative course was uncomplicated, the patient’s dysphagia improved, and reflux esophagitis did not worsen.

Treating refractory achalasia can be challenging. LHM and POEM are both considered first-line treatments. Nowadays, the treatment outcomes for achalasia patients are dichotomized according to the Eckardt score, which defines success as an Eckardt score ≤ 3 [8]. However, even in cases defined as successful based on their Eckardt score, a patient’s subjective symptoms can be sufficiently discomforting for them to seek additional treatment, as in the presently reported case. The treatments in such cases are considered clinical failures.

As initial treatments, the success rates of both LHM and POEM are sufficiently high (81.7% for LHM, 83% for POEM) [5]. Nevertheless, certain patients may experience clinical failure. Several studies have reported the success rates and adverse effects of different secondary treatments after unsuccessful POEM or LHM (Table 1) [9–23]. Unsuccessful LHM is generally followed by POEM because of the lower degree of invasiveness, and these secondary treatments have a high likelihood of success. On the other hand, the success rate after retreatment following an unsuccessful POEM ranges from 29 to 100%, making retreatment controversial. The major reasons for primary failure of LHM or POEM are incomplete myotomy and undesirable sclerosis in the vicinity of the myotomy site. Zaninotto et al. reported incomplete myotomy or sclerosis, especially at the distal site of the myotomy as the main reason for the failure of LHM [24]. On the other hand, to the best of our knowledge, the major etiology and location of the incomplete myotomy in patients with failure of POEM remain under debate. The reasons for failure of POEM are possibly more complex than those underlying failure of LHM which could somehow influence the success rate of following secondary treatment. Variations in the results of POEM may be associated with the endoscopic approach. In our case, we obtained a more careful history of the patient’s symptoms and conducted a thorough clinical examination to better understand the pathology underlying the failure of POEM. Our patient had significant type 1 achalasia and morbid reflux. We considered that performing a myotomy as deep as possible with mild anti-reflux fundoplication would be the most suitable treatment for our patient, which indeed did work well.

**Table 1:** Success rate and adverse event of secondary treatment for refractory achalasia

1st and 2nd treatment	Author	Year	Reference	Eckardt score		Success rate (%)	Cohort size	Adverse events
				Before 2nd treatment	After 2nd treatment			
LHM → LHM	Vigneswaran	2016	9	5.33	1	N/A	3	Perforation, mediastinal abscess: 33.3%
LHM → POEM	Zhou	2013	10	9.2	1.3	92	12	Postoperative: GERD:8.3%
	Vigneswaran	2016	9	6.8	0.6	N/A	5	Persistent subcutaneous emphysema: 20%
	Ngamruengphong	2017	11	7.1	2.09	81%	90	Mucosotomy: 3.3%
								Delayed bleeding: 1.1%
								Subcutaneous emphysema: 1.1%
								Submucosal hematoma: 1.1%
								Pneumonia: 1.1%
								Mediastinitis: 1.1%
	Landi	2017	12	6.4	1.9	79	14	Postoperative GERD: 50%
	Kristensen	2017	13	6.75	4.25	N/A	14	N/A
	Zhang	2018	14	7.25	1.5	96	46	N/A
	Tyberg	2018	15	7.98	1.72	94	51	Mucosal defect: 11.7%
	Sanaka	2018	16	6.83	0.67	N/A	29	Mucosal perforation: 11.7%
								Mediastinitis: 4.0%
	Arshava	2018	17	5	2.5	N/A	4	N/A
	Huang	2021	18			90	272	Postoperative symptomatic reflux: 36.9%
								Postoperative endoscopically proven esophagitis: 33.0%
								Postoperative pH monitoring based acid exposure: 47.8%
POEM → POEM	Li	2015	19	4.3	1	100	15	Submucosal tunnel infection: 7%
	Tyberg	2017	20	4.3	1.64	85	46	Peri-procedural bleeding: 17.0%
	van Hoeij	2018	21			63	8	None
	Ichkhanian	2021	22	6.1	2.1	76	33	Esophageal leak: 3.0%
								Symptomatic pneumoperitoneum: 3.0%
								Subcutaneous emphysema: 3.0%
								Inadvertent mucosotomy: 3.0%
POEM → LHM	Giulini	2017	23	NA	NA	100	1	None
	van Hoeij	2018	21	NA	NA	45	11	None
	Ichkhanian	2021	22	6.9	4	29	7	None

Van Hoeij reported 11 cases of LHM after unsuccessful POEM. The success rate was 45%, which was significantly superior to that of pneumatic dilation (22%) but not significantly different from that of repeat POEM (63%). Ichkhanian et al. reported 7 cases of secondary LHM after unsuccessful POEM with a success rate of 29%, demonstrating a statistical difference against repeat POEM (76%). The LHM-treated patients in this previous study tended to have severe conditions, which might have affected the results. Data regarding the treatment of unsuccessful POEM are scarce, making customized medical treatment plans necessary for each patient.

LHM with Dor fundoplication has some merit, compared with POEM. While POEM tends to fail because of an incomplete myotomy [25, 26], laparoscopic myotomy is performed under a magnified field of vision, making it easier to control the length and depth of incisions. In our case, we performed more complete dissection with rather loose Dor fundoplication than is usually undertaken. We would like to emphasize the higher controllability of cutting the muscles and fibers causing the symptoms in LHM. Also, the addition of fundoplication is an advantage of LHM with Dor fundoplication. LHM with Dor fundoplication is known to have a lower risk of postoperative GERD than POEM (15.2% vs. 37.4%) [27]. Not only POEM but also repeated POEM is associated with a relatively high incidence of postoperative GERD (33.3%) [19]. Thus, LHM with Dor fundoplication has the advantage of enabling a direct field of vision and allowing the tightness of the fundoplication to be adjusted, which might lower the risk of postoperative GERD.

Addition of an appropriate fundoplication procedure (Dor, Nissen, or Toupet techniques) is essential for improving the outcome of LHM. The Dor procedure involves partial wrapping of the anterior wall, the Nissen procedure consists of full wrapping of the posterior wall, and the Toupet procedure consists of partial wrapping of the posterior wall. Among the three, the Dor procedure is the preferred method with LHM. Rebecchi et al. reported that Dor fundoplication resulted in a significantly lower rate of postoperative dysphagia than Nissen fundoplication, presumably as partial wrapping would ensure proper opening of the cardia [28]. As for comparison between the two partial wrapping procedures, Dor and Toupet, an RCT of 73 patients reported better outcomes of the Dor procedure. Eckardt scores of < 3 were obtained in 100% of subjects who underwent Dor fundoplication and 90% of patients who underwent the Toupet procedures at 24 months [29]. It is possible that the anterior wall wrapping in the Dor procedure guarantees sturdiness by protecting the mucosa at the incision site, allowing more complete myotomy. On the other hand, conversely, postoperative GERD tends to be better controlled by Nissen > Toupet > Dor [30–32]. In light of the need to achieve reliable improvement in the dysphagia, as in our case, and the expectation that pharmacotherapy can also be useful to control GERD, we encourage surgeons to choose the Dor procedure.

For these reasons, we propose that LHM with Dor fundoplication should be performed proactively in patients with unsuccessful POEM.

## Conclusion

LHM with Dor fundoplication seems to be safe and critically effective even for patients with refractory achalasia and a history of unsuccessful POEM since it has the advantages of allowing a sufficient myotomy and providing additional anti-reflux surgery.

## Data Availability

The data used in this study are available from the corresponding author upon reasonable request.

## Abbreviations

AET: Acid exposure time
GERD: Gastroesophageal reflux disease
HRM: High-resolution manometry
IRP: Integrated relaxation pressure
LES: Lower esophageal sphincter
LHM: Laparoscopic Heller myotomy
POEM: Peroral endoscopic myotomy
TBE: Timed barium esophagogram
